# Age of the oldest known *Homo sapiens* from eastern Africa

**DOI:** 10.1038/s41586-021-04275-8

**Published:** 2022-01-12

**Authors:** Céline M. Vidal, Christine S. Lane, Asfawossen Asrat, Dan N. Barfod, Darren F. Mark, Emma L. Tomlinson, Amdemichael Zafu Tadesse, Gezahegn Yirgu, Alan Deino, William Hutchison, Aurélien Mounier, Clive Oppenheimer

**Affiliations:** 1 Department of Geography, University of Cambridge, Cambridge, UK; 2 Fitzwilliam College, University of Cambridge, Cambridge, UK; 3 School of Earth Sciences, Addis Ababa University, Addis Ababa, Ethiopia; 4 Department of Mining and Geological Engineering, Botswana International University of Science and Technology, Palapye, Botswana; 5 NEIF Argon Isotopes, University of Glasgow, SUERC, Glasgow, UK; 6 Trinity College Dublin, University of Dublin, Dublin, Ireland; 7 Department of Geosciences, Environment and Society, Université Libre de Bruxelles, Brussels, Belgium; 8 Berkeley Geochronology Center, Berkeley, CA USA; 9 School of Earth and Environmental Sciences, University of St Andrews, St Andrews, UK; 10 Histoire Naturelle de l’Homme Préhistorique (HNHP, UMR 7194), MNHN/CNRS/UPVD, Musée de l’Homme, Paris, France; 11 Leverhulme Centre for Human Evolutionary Studies, Department of Archaeology, University of Cambridge, Cambridge, UK; 12 McDonald Institute for Archaeological Research, Cambridge, UK

**Keywords:** Archaeology, Geochemistry, Volcanology

## Abstract

Efforts to date the oldest modern human fossils in eastern Africa, from Omo-Kibish^1–3^ and Herto^4,5^ in Ethiopia, have drawn on a variety of chronometric evidence, including ^40^Ar/^39^Ar ages of stratigraphically associated tuffs. The ages that are generally reported for these fossils are around 197 thousand years (kyr) for the Kibish Omo I^3,6,7^, and around 160–155 kyr for the Herto hominins^5,8^. However, the stratigraphic relationships and tephra correlations that underpin these estimates have been challenged^6,8^. Here we report geochemical analyses that link the Kamoya’s Hominid Site (KHS) Tuff^9^, which conclusively overlies the member of the Omo-Kibish Formation that contains Omo I, with a major explosive eruption of Shala volcano in the Main Ethiopian Rift. By dating the proximal deposits of this eruption, we obtain a new minimum age for the Omo fossils of 233 ± 22 kyr. Contrary to previous arguments^6,8^, we also show that the KHS Tuff does not correlate with another widespread tephra layer, the Waidedo Vitric Tuff, and therefore cannot anchor a minimum age for the Herto fossils. Shifting the age of the oldest known *Homo sapiens* fossils in eastern Africa to before around 200 thousand years ago is consistent with independent evidence for greater antiquity of the modern human lineage^10^.

## Abstract (web summary)

Geochemical analyses correlating the stratum that overlies the sediments containing the Omo fossils with material from a volcanic eruption suggest that these fossils (the oldest known modern human fossils in eastern Africa) are over 200,000 years old.

## Main

Only eight sites in Africa have yielded possible early anatomically modern *Homo sapiens* fossils from the late Middle Pleistocene (approximately 350–130 thousand years ago (ka))^11^. Most of these have considerable age uncertainty or debatable *H. sapiens* apomorphy^11^. A principal method for constraining the fossil ages is the use of single-crystal ^40^Ar/^39^Ar isotope dating applied to stratigraphically associated volcanic ash (tephra) beds^12–14^. However, many distal tephra deposits consist largely of glass and lack suitable crystals for dating. In this case, geochemical fingerprinting can be used to match a tephra layer to more readily dated proximal deposits with larger, more abundant phenocrysts. The most widely accepted fossils that are interpreted as possessing unequivocal modern cranial apomorphies (that is, a tall cranial vault and a chin) and classified as *H. sapiens* are two Ethiopian finds^11,15,16^, namely the Omo I^1^ and Herto specimens^4^. Accordingly, the evidence that constrains their ages assumes particular importance but is a topic of considerable geochronological controversy^3,6,8^.

**Fig. 1:**
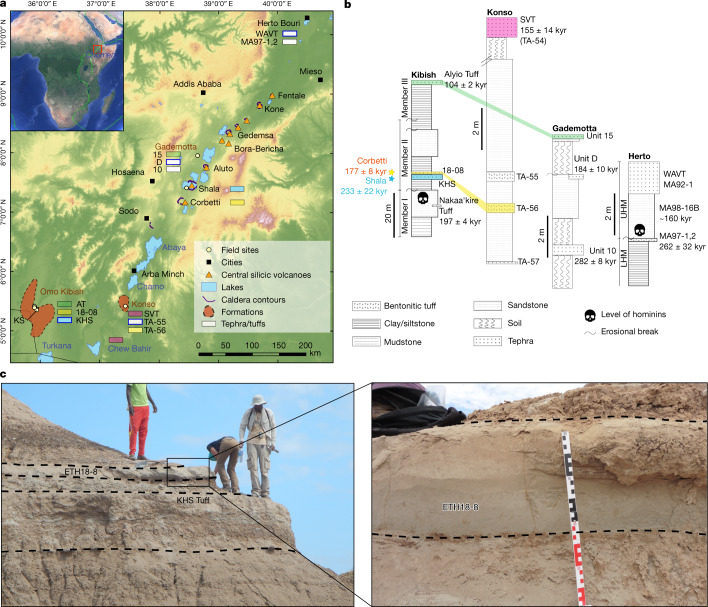
Late Middle Pleistocene tephrostratigraphy of the Main Ethiopian Rift. **a**, Map of the MER showing silicic volcanoes and the late Middle Pleistocene sedimentary formations and relevant tephra units. White boxes with blue edges depict former correlatives of the KHS Tuff^6,8^ **b**, Synthetic stratigraphic logs of the late Middle Pleistocene formations showing former correlations for the Alyio Tuff^6^ (green), Konso SVT (pink, also identified in the Chew Bahir sediment core^33^), new correlations for Konso unit TA-56 (yellow), and source eruptions (stars). LHM, lower Herto Member; UHM, upper Herto Member. **c**, Tephra ETH18-8 above the KHS Tuff at the KS locality in the Omo-Kibish Formation^9^.

The Omo I remains were discovered in the late 1960s in the lower Omo valley of southern Ethiopia^1,14^, at the surface of a siltstone near the top of Member I of the Omo-Kibish Formation (Fig. 1a, b). The maximum age of Omo I was derived from the ^40^Ar/^39^Ar age of 196 ± 4 kyr (2*σ*)^3,6,17^ obtained for alkali feldspar phenocrysts from the three youngest pumice clasts that were sampled from a heterogeneous tuffaceous deposit correlated with the Nakaa’kire Tuff^3^, which is reported to lie “near, but probably slightly below” the fossils^3^ (Fig. 1b). Recalculated using a more widely adopted age of 28.201 million years (Myr) for the irradiation monitor (sanidine from the Fish Canyon Tuff of Colorado)^18^, the Nakaa’kire Tuff age shifts marginally to 197 ± 4 kyr. Owing to the uncertain stratigraphic relationship between this tuff and the hominin fossils^19^, much attention has been focused on dating the KHS Tuff—a widespread, more-than-2-m-thick deposit of fine ash fallout at the base of Member II of the Omo-Kibish Formation (Fig. 1b). The KHS Tuff overlies Member I, where Omo I was retrieved around 1.4 m lower down section, and is demonstrably younger than the fossils^3,9^. Although the Nakaa’kire Tuff was identified in several sections below the KHS Tuff, the latter was not found in the same section from which the dated pumice clasts correlated with the Nakaa’kire Tuff (on the basis of major element composition) were sampled^3^. The fine grain size of the KHS Tuff has precluded direct ^40^Ar/^39^Ar dating, and no correlation to a source volcano or proximal pyroclastic unit has to our knowledge been made previously. However, drawing on published major element glass compositions, it has been correlated with both tephra TA-55^20,21^ from the Konso Formation and the directly ^40^Ar/^39^Ar-dated 184 ± 10 kyr unit D^22^ (recalculated age) of the Gademotta Formation^6^ (Fig. 1b). Relating the sediment flux in the Omo-Kibish basin with high lake levels that correspond to Mediterranean sapropel deposition^9,23^, a slightly younger age for the KHS Tuff of around 172 kyr has also been proposed^6^. Either of these ages (184 or 172 kyr) would be consistent with the proposed age of 197 ± 4 kyr for Omo I.

The Herto *H. sapiens* fossils were recovered in the late 1990s in the Middle Awash^4,5^ (Afar rift; Fig. 1a). They were preserved in a sandstone within the upper Herto Member of the Bouri Formation (Fig. 1b). This sandstone is capped by the Waidedo Vitric Tuff (WAVT) (Fig. 1b), which is widespread across western Afar and is also present at Gona^24^, 50 km north of Herto. Direct dating of the WAVT has remained inconclusive owing to crystal contamination, but dating of pumice and obsidian clasts in the fossiliferous sandstone yielded a maximum age of around 160 kyr (ref. ^5^). The WAVT was identified as a distal correlative of tephra TA-55 (Fig. 1b), on the basis of major element analysis of individual grains and major and trace element analysis of purified bulk separates^5,25^. In Konso, unit TA-55 lies below the 155 ± 14 kyr Silver Tuff^5^ (SVT) (recalculated age) (Fig. 1b), suggesting an age for the Herto fossils of around 160–155 kyr (ref. ^4^). This finding was challenged, however, in a study^6^ that correlated the Kibish KHS with Konso TA-55, and therefore with the Herto WAVT (Fig. 1b). This argument suggested an age of around 172 kyr for the WAVT, contradicting the established Herto stratigraphy. The Herto research group^8^ responded by corroborating their original stratigraphy, with the WAVT above the Herto fossils, thus challenging an age of about 172 kyr for the KHS. They concluded that the KHS, Konso unit TA-55^5^, Gademotta unit D (around 184 kyr)^22^ and WAVT^5^ could all represent a single tephrostratigraphic marker lying above the Omo-Kibish and Herto *H. sapiens* fossils, but that multiple eruptive sources would also be plausible^8^ (Fig. 1b). Given the lingering uncertainties of the stratigraphic relationship of the Nakaa’kire Tuff to Omo I, the age of the KHS Tuff becomes critical to the chronostratigraphy of these sites.

We have re-sampled the KHS Tuff and other pertinent ash deposits at Omo-Kibish, Konso and Gademotta to assess the geochemical correlations from which the ages of the oldest modern human fossils are inferred. While revisiting the sampling locality of the KHS Tuff (KS type section)^9^ at Omo-Kibish, we sampled another tephra layer in Member II (Fig. 1c) in an outcrop about 100 m from the KS type section. Unit ETH18-8 is an approximately 15-cm-thick, very well-sorted crystal-rich fine sand grey tephra layer situated 40 cm above the KHS Tuff (Fig. 1c). It is ubiquitous between the KHS section (KS) and the Chibele section (CB), and might stratigraphically correspond to unit CRF-23 previously identified above the KHS Tuff at the CB section^9^, although this cannot be confirmed through geochemical analysis because of the different microprobe conditions used.

**Fig. 2:**
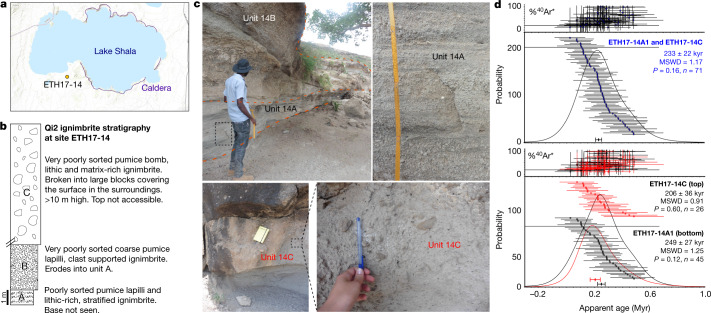
Stratigraphy and age of the Shala Qi2 ignimbrite. **a**, Location of site ETH17-14 near Lake Shala in the MER. **b**, Synthetic stratigraphy of the Qi2 ignimbrite of Shala at location ETH17-14. **c**, Photographs of units 14A, 14B and 14C of the Qi2 ignimbrite at site ETH17-14. Field observations indicate that deposits 14A and 14B are subunits of the same phase of the Qi2 eruption. **d**, ^40^Ar/^39^Ar age pooled data plotted on ideograms for samples 14A and 14C of the Qi2 ignimbrite (bottom) yielding a preferred composite eruption age of 233 ± 22 kyr (top). Data are weighted means. Error bars show data and results at 2*σ*. ^40^Ar*, radiogenic ^40^Ar; MSWD, mean square of weighted deviates; *P*, probability that residuals are explained by measurement errors exclusively; *n*, number of accepted grains.

In an attempt to identify and date the eruption that generated the KHS tuff, we included samples of ignimbrites from the caldera-forming eruptions of Shala and Corbetti volcanoes. Shala and Corbetti are the only Main Ethiopian Rift (MER) systems known to have produced major eruptions between around 170 ka and 250 ka^26^. At Shala, the largest caldera in the central MER (Fig. 2a), we sampled at a more-than-20-m-thick exposure of the unwelded Qi2 ignimbrite^27^ (Fig. 2b, c), southwest of Lake Shala and 350 km northeast of Omo-Kibish (Fig. 2a). We also analysed glass from a welded ignimbrite (COI2E) attributed to the formation of Corbetti caldera, dated at 177 ± 8 kyr (ref. ^26^). A challenge of geochemical correlations between proximal and distal tephra deposits in the region is similarity in major and trace element compositions between pyroclastic products, not only of the same volcano but of different volcanoes in the MER^28^. Accordingly, correlations are ideally based on a detailed suite of major, minor and trace element single-grain glass shard or pumice glass analyses.

**Fig. 3:**
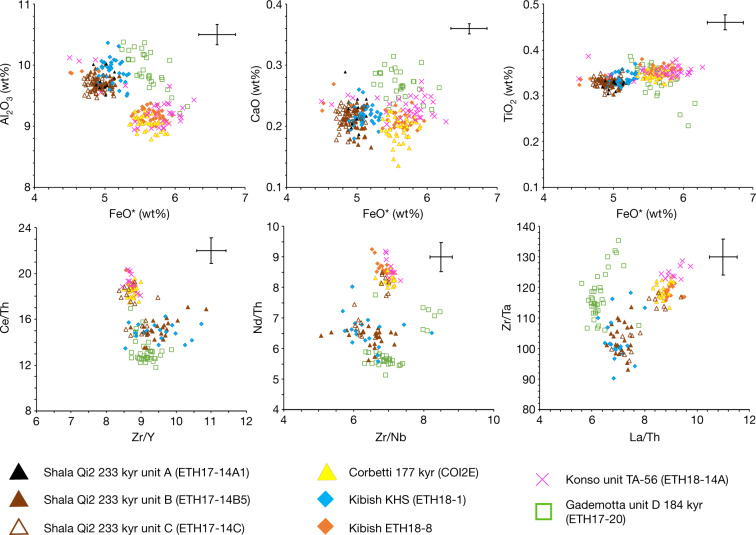
Geochemical fingerprints of MER tephra and their sources. Major element abundances and trace element ratios of glasses from the Shala Qi2 ignimbrite (around 233 kyr), the Corbetti ignimbrite (around 177 kyr), the Gademotta unit D (around 184 kyr), the Kibish KHS and ETH18-8 tuffs, and the Konso TA-56 tuffs (all data from this study). Major element data are normalized to 100% anhydrous. Error bars shown are relative standard deviations derived from repeat measurements of matrix match glass secondary standards STH-S6 (for FeO*, *n* = 91; Supplementary Table 6) and ATHO-G (for Al_2_O_3_, CaO and TiO_2_, *n* = 70; Supplementary Table 6). They are plotted in the top right corner of each plot for clarity and rescaled to the value of the centre point. In the case of element ratios, error propagation has been applied using analyses of standard ATHO-G (*n* = 15; Supplementary Table 7). Additional compositional observations and biplots are presented in Supplementary Fig. 5.

The KHS glass shards are homogeneous pantelleritic rhyolite in composition (77.0 ± 0.3 wt% SiO_2_, 9.7 ± 0.1 wt% Al_2_O_3_, 5.0 ± 0.1 wt% FeO* (FeO* refers to the total Fe as FeO) and 7.1 ± 0.4 wt% Na_2_O+K_2_O; Supplementary Table 1). Immobile oxide abundances, including FeO*, CaO, Al_2_O_3_ and TiO_2_ (Fig. 3, Supplementary Table 1), correspond with those of glasses from the proximal products of the Qi2 eruption of Shala volcano (samples ETH17-14A1, B1, B5 and C) (Figs. 2b, c, 3, Supplementary Fig. 4, Supplementary Table 1, Supplementary Information). These correlations are corroborated by comparing immobile trace element ratios for Qi2 and KHS glasses and principal component analysis (Fig. 3, Supplementary Figs. 4, 5, Supplementary Table 2, Supplementary Information).

In addition, we find that the COI2E pantelleritic rhyolite glass from the 177 ± 8 kyr (ref. ^26^) Corbetti ignimbrite (74.3 ± 0.2 wt% SiO_2_, 9.1 ± 0.1 wt% Al_2_O_3_, 5.6 ± 0.2 wt% FeO* and 10.1 ± 0.2 wt% Na_2_O+K_2_O) (Fig. 3, Supplementary Fig. 4, Supplementary Table 1, Supplementary Information) has immobile oxides and trace element abundances that match those for Kibish unit ETH18-8 and Konso TA-56 (Fig. 3, Supplementary Figs. 4, 5, Supplementary Table 2, Supplementary Information).

We used the ^40^Ar/^39^Ar dating method to analyse 113 individual sanidine crystals extracted from pumice samples ETH17-14A1 (base, 68 crystals) and ETH17-14C (top, 45 crystals) collected from the Shala Qi2 deposits (Fig. 2). The resulting data were filtered to exclude grains with low gas yields, at or below blank level, and xenocrysts with ages significantly older than the mean of the dataset (six grains with ages exceeding 1 Myr). The distributions of ages from each sample were indistinguishable at 2*σ* uncertainty (Fig. 2d). Combining analyses from both pumice samples yielded a weighted mean of 233 ± 22 kyr at 2*σ* (Fig. 2d, Supplementary Table 3), thereby dating the Qi2 eruption and the KHS tuff.

An age of 233 ± 22 kyr for KHS is consistent with the 177 ± 8 kyr age that we associate with the overlying ETH18-8 tephra (Fig. 1b). However, it casts doubt on the suggested correlation between high deposition fluxes in the Omo basin with large in-flows of fresh water from the Nile River system into the Mediterranean sea^6,7,9^, at least during the formation of Member II. Our KHS age is incongruent with the formation of Mediterranean Sapropel S6 at 172 ka^6^, and instead overlaps the timing of the formation of sapropel S8 (217 ka)^9,29^. Although the 177 ± 8 kyr age of ETH18-8 is consistent with the formation of sapropel S6 (172 ka)^29^, only a mudstone unit of around 40 cm thickness separates KHS from ETH18-8, which cannot account for the suggested rapid deposition in the basin concomitant with sapropel S7 (192–199 ka)^3^.

The revised Omo-Kibish stratigraphy is also incompatible with the 197 ± 4 kyr age reported for the Nakaa’kire Tuff^3,7,9^, which is found in Member I of the formation^3,7,9^ and which must therefore be older than 233 ± 22 kyr. The age of 197 ± 4 kyr was inferred from three out of five dated pumice clasts from lenses found in ‘a sandy tuffaceous matrix’^7^. Although these samples had similar major element compositions to the Nakaa’kire Tuff, they were collected from a lateral outcrop and not in section^3,7,9^. Given the uncertainty in the age and stratigraphic placement of the Nakaa’kire Tuff, as well as its heterogeneous lithology and geochemistry, the identification of the 233 ± 22 ka Qi2 eruption of Shala as the source of the KHS Tuff provides a more robust minimum age for Omo I *H. sapiens*.

Furthermore, our glass compositional data, source correlation and age estimate for KHS allow us to re-assess its identification at other archaeological sites in Ethiopia. New lithological examination of the pedogenically altered unit TA-55 at Konso (Supplementary Fig. 1) in grain size fractions of greater than 125 µm, greater than 80 µm and greater than 25 µm, after density separation, failed to identify glass shards in this deposit, which was previously correlated with the WAVT at Herto. This precluded evaluation of the reported correlation with the KHS Tuff^6^. However, with the underlying unit TA-56 now correlated with Kibish unit ETH18-8 and the 177 ± 8 kyr Corbetti ignimbrite (Fig. 3, Supplementary Figs. 4, 5), it is clear that TA-55 is younger than 177 ± 8 kyr and so cannot correlate with Qi2 or the KHS Tuff.

Although the 184 ± 10 kyr unit D of Gademotta appears close to KHS in major element contents, neither major nor trace element abundances clearly overlap (Fig. 3, Supplementary Figs. 4, 5, Supplementary Information), precluding a match. Immobile trace element ratios and principal component analysis show that unit D also differs from TA-56 (Fig. 3, Supplementary Figs. 4, 5, Supplementary Information).

The correlation of the Herto WAVT with Konso unit TA-55^5^, around 800 km south of Herto, led earlier investigators to accept the 155 ± 14 kyr age of the SVT at Konso as the *terminus ante quem* of the Herto fossils. This correlation has been debated^30^ but reinforced by additional geochemical data^25^. We were unable to find preserved glass in our TA-55 sample but our results undermine the tephrostratigraphic correlations proposed between the Omo-Kibish, Gademotta and Konso formations^6^ and bracket the age of the Konso TA-55 tuff between 177 ± 8 kyr (TA-56) and 155 ± 14 kyr (SVT). Although its correlation with the WAVT at Herto should be confirmed in the future using grain-discrete single-point glass analyses, this age bracket is consistent with the underlying Herto fossiliferous sandstone (approximately 160 kyr)^5^, and confirms that the Herto *H. sapiens* fossils are considerably younger than Omo I at Omo-Kibish.

Our new age constraints are congruent with most models for the evolution of modern humans, which estimate the origin of *H. sapiens* and its divergence from archaic humans at around 350–200 ka (refs. ^16,31,32^). The challenge remains to obtain a robust maximum age for Omo I. Our revised tephrostratigraphy demonstrates that the Herto specimens postdate the Omo I remains from Omo-Kibish, and that they do not lie beneath the same tephra horizon as the Kibish fossils, as previously inferred^8^. Further geochemical data are needed to clarify the relationship between the WAVT and other MER tephra, and may ultimately identify the WAVT source, promising a more reliable minimum age for the Herto fossils. More generally, continued efforts to develop the tephrochronological framework for eastern Africa will help in addressing a range of interrelated volcanological, palaeoenvironmental and palaeoanthropological questions.

## Methods

### Sampling

Stratigraphic descriptions and sampling were carried out during two field seasons in 2017 and 2018. We sampled the previously described^27^ Qi2 eruption of Shala volcano, and we revisited the Konso^20,21^, Omo-Kibish^3,6,9^ and Gademotta^22,34^ formations (Fig. 1). At each site we described extensively the stratigraphy of the outcrops, measured the thickness of units and sampled deposits where best exposed and least altered.

### ^40^Ar/^39^Ar dating

Feldspars were extracted from pumice samples at the Departments of Geography and Earth Sciences, University of Cambridge. Rocks were crushed in a jaw crusher and sieved to obtain a 250–500-μm size fraction, cleaned under water and passed through a Frantz magnetic barrier laboratory separator to isolate sanidine phenocrysts from the groundmass. Because separates would still contain other phases (primarily glass and quartz), 100–200 sanidine grains were further handpicked and then leached in 5% HF to remove any glass attached to the crystals.

Samples and neutron flux monitors were packaged in copper foil and stacked in quartz tubes with the relative positions of packets precisely measured for later reconstruction of neutron flux gradients. The sample package was irradiated for 2 h in the Oregon State University reactor, Cd-shielded facility (CLICIT). Fish Canyon sanidine (28.294 ± 0.036 (1*σ*) million years ago; Ma) (ref. ^35^) was used to monitor ^39^Ar production and establish neutron flux values (*J*) for the samples (Supplementary Table 4). Gas was extracted from samples via step-heating using a mid-infrared (10.6 µm) CO_2_ laser with a non-gaussian, uniform energy profile and a 1.5-mm beam diameter. The samples were housed in a doubly pumped ZnS-window laser cell and loaded into a stainless steel planchette containing 208 2.0-mm-diameter round wells. Liberated argon was purified of active gases—for example, CO_2_, H_2_O, H_2_, N_2_ and CH_4_—using three Zr-Al getters; one at 16 °C and two at 400 °C. Data were collected on a Mass Analyser Products MAP-215-50 single-collector mass spectrometer using an electron multiplier collector in dynamic collection (peak hopping) mode. Time-intensity data were regressed to inlet time with second-order polynomial or linear fits to the data. Sample runs were corrected using the standard deviation of blanks throughout the runs. Mass discrimination was monitored on a daily basis, between and within sample runs by analysis of an air standard aliquot delivered by an automated pipette system (see Supplementary Table 4 for *D* values). All blank, interference and mass discrimination calculations were performed with the MassSpec software package (MassSpec, v.8.058, A. Deino, Berkeley Geochronology Center). Decay constants and corrections (Supplementary Table 5) were made using the approach of Renne et al. 2010^36^ with the parameters of Renne et al. 2011^35^.

Following the approach of Kuiper et al. ^18^, samples with low radiogenic yields (^40^Ar* < 10%, 23 grains), and obvious outliers (age > 1 Myr, 6 grains) were rejected. After this initial filtering, peak age distributions were defined by determining the youngest population of individual grain analyses (*n* ≥ 10) that conforms to a Gaussian distribution with the expected scatter as indicated by the value of the mean square of weighted deviates (MSWD)); this second stage of filtering resulted in the rejection of an additional ten older grains, leaving 71 accepted grains.

Ages for unit samples ETH17-14A1 and ETH17-14C are reported with two sigma errors in Supplementary Table 3 with the raw data in Supplementary Table 4. These two sub-samples from the top and bottom of the same stratigraphic unit are indistinguishable in age at 2*σ* uncertainty, which permits them to be combined into a single composite sample. The accepted age for this population is 234 ± 22 kyr (relative to ref. ^36^) or 233 ± 22 kyr (relative to ref. ^18^). An inverse isochron plotted through the data (Supplementary Fig. 2) yields an age of 219 ± 27 kyr (^40^Ar/^36^Ar_(i)_ = 314 ± 24, MSWD = 1.1, *P* = 0.19, *n* = 71), which is indistinguishable from the accepted age.

Although we are using the Kuiper et al. (ref. ^18^) calibration, the Renne et al. 2011 (ref. ^36^) calibration has quantifiable uncertainties and is our preferred age for the sample. Nevertheless, for consistency with previous work, the latter age (233 ± 22 kyr) is used throughout the manuscript.

### Sample preparation for geochemical analyses

Sample preparation was carried out in the Cambridge Tephra Laboratory in line with the protocols of the International Focus Group on Tephrochronology (INTAV)^12,37^ for geochemical characterization of volcanic glass. Pumice samples of the Qi2 Shala eruption were crushed, sieved at 500, 250, and 125 μm, and washed in purified water and hydrochloric acid (1%) in an ultrasonic bath. Glass grains from the 125–250-μm fraction were handpicked under microscope, mounted in epoxy resin stubs, then sectioned and polished. Distal tephra samples from Gademotta (unit D), Konso (TA-55/ETH18-14B and TA-56/ETH18-14A) and Omo-Kibish formations (KHS, ETH18-08) were washed through a sieve in purified water at 80 or 25 μm, then dried, described under microscope and mounted in epoxy resin stubs, then sectioned and polished. Strongly altered samples of TA-56 (ETH18-14A) and TA-55 (ETH18-14B) units from the Konso formation were density extracted to facilitate the search for volcanic glass^38,39^. Sample ETH18-14B from TA-55 was sieved at 125, 80 and 25 μm and residues inspected under the microscope, yet no glass was found.

### Major element analysis

Mounted samples were analysed for major element compositions with a SX100 CAMECA electron microprobe at the Department of Earth Sciences, University of Cambridge. Major elements were measured with an accelerating voltage of 10 kV and a 10-nA defocused beam. Elements were counted on-peak for 10 s (Na, Si), 20 s (Al, Fe and K), 60 s (Ti, Mg, Ca, and Cl), 90 s (P) and 120 s (Mn). Sodium was measured first to minimize alkali loss. The analytical accuracy was checked against international standards ATHO-G, STH-S6 and internal peralkaline obsidian from Lipari (74 wt% SiO_2_, 3.8 wt% Na_2_O and 5.3 wt% K_2_O). Replicate standard analyses and standard deviations are reported in Supplementary Table 6. The latter are used for error bars on biplots instead of the standard deviation of each sample, which is affected by their natural variability. Where possible, we analysed 40–50 points per sample. All analyses are reported in Supplementary Table 1.

### Trace element analysis

Trace element compositions of individual tephra shards were analysed by laser ablation inductively coupled plasma mass spectrometry (LA-ICP-MS) at the iCRAG laboratory at Trinity College Dublin. The instrument used was a Thermo iCAPQ coupled to a Photon Machines 193-nm G2 laser and a Helex two-volume cell. We used a spot size of 40 µm, depending on the area available for analysis, a repetition rate of 6 Hz and a count time of 33 s (200 pulses) on the sample and 30 s on the gas blank (background). We analysed large-enough glass shards analysed by electron microprobe analysis (EMPA) for major elements; however, spots are not tied through codes as we used the average Ca concentration of each sample as Ca correction factor. Concentrations were calibrated using NIST612 with ^29^Si as the internal standard. Data reduction was undertaken in Iolite v.3.4 and a secondary Ca correction factor was applied^40^. Accuracies of ATHO-G and StHs6/80-G MPI-DING glass analyses are typically better than 6% for most elements. The precision is reflected by the standard deviations of replicate standard analyses (Supplementary Table 7), used for error bars on Fig. 3, Supplementary Fig. 4. Standard deviations of trace element ratios (Fig. 3) take into account error propagation. Detailed compositions of samples are reported in Supplementary Table 2.

### Reporting summary

Further information on research design is available in the Nature Research Reporting Summary linked to this paper.

## Online content

Any methods, additional references, Nature Research reporting summaries, source data, extended data, supplementary information, acknowledgements, peer review information; details of author contributions and competing interests; and statements of data and code availability are available at 10.1038/s41586-021-04275-8.

## Supplementary information

Supplementary Information This file contains information on tephra geochemistry, Supplementary Figs. 1–5, and additional references.

Reporting Summary

Supplementary Table 1 Major element normalized composition of tephra samples.

Supplementary Table 2 Trace element abundances (ppm) of tephra samples.

Supplementary Table 3 Single-crystal ^40^Ar/^39^Ar ages for unit A and unit C of the Qi2 ignimbrite of Shala.

Supplementary Table 4 Argon isotopic data for the Qi2 samples.

Supplementary Table 5 Decay constants and correction factors.

Supplementary Table 6 Average compositions of EPMA secondary standards for years 2018–2019.

Supplementary Table 7 Standard compositions for LA-ICP-MS analyses.

## Data Availability

All data supporting the findings of this study are available within the paper and its Supplementary Information files. Background maps for Fig. 1 are Shuttle Radar Topography Mission Digital Elevation Model data at one arcsecond resolution from the NASA Land Processes Distributed Active Archive Center (https://earthexplorer.usgs.gov/); settlements, lakes and other features are from (https://www.naturalearthdata.com/). Background image for the top left corner inset of Fig. 1 from Google Earth and plate boundaries data courtesy of the US Geological Survey.
