# Supplementary material for: Age of the oldest known Homo sapiens from eastern Africa
Source: Nature. 2022 Jan 12;601(7894):579–83. doi: 10.1038/s41586-021-04275-8 (PMC8791829; doi:10.1038/s41586-021-04275-8)
Supplement: Supplementary file 1 — This file contains information on tephra geochemistry, Supplementary Figs. 1–5, and additional references. [file 41586_2021_4275_MOESM1_ESM.pdf]

nature portfolio

https://doi.org/10.1038/s41586-021-04275-8

Supplementary information

Age of the oldest known *Homo sapiens* from eastern Africa

In the format provided by the authors and unedited

Age of the oldest *Homo sapiens* from eastern Africa

Céline M. Vidal[1,2*], Christine S. Lane[1], Asfawossen Asrat[3,4], Dan N. Barfod[5], Darren F. Mark[5], Emma L. Tomlinson[6], Amdemichael Zafu Tadesse[7], Gezahegn Yirgu[3], Alan Deino[8], William Hutchison[9], Aurélien Mounier[10,11], Clive Oppenheimer[1,12]

Corresponding author: Céline Vidal cv325@cam.ac.uk

SUPPLEMENTARY INFORMATION CONTENTS

Supplementary Tables are in a separate file

Tephra Geochemistry - pages 2-5

Supplementary Figures - pages 6-10

Figure S1 – page 6

Figure S2 – page 7

Figure S3 – page 8

Figure S4 – page 9

Figure S5 – page 10

TEPHRA GEOCHEMISTRY

Introduction

There are only minor geochemical differences between MER silicic tephra (e.g. ref.[1]), highlighting the importance of combining evidence from the volcanological record and the chronostratigraphy of distal sequences (the latter point is discussed in the main text) when characterising and correlating samples. Our study includes proximal samples from Corbetti and Shala, which are the only MER systems known to have produced major eruptions between ~170 ka and ~250 ka (ref.[2]), the timeframe relevant for the tuffs considered here. The volumes of silicic magma associated with the caldera formation of Shala (86-170 km^3) and Corbetti (25-63 km^3) are by far the largest among MER volcanoes[2]. Tephra from the major eruptions of these two volcanoes are thus the most likely to be preserved within distal sedimentary formations.

Our set of distal tephra focused on tuffs from the time interval relevant to the dating of KHS and Omo I fossils at Kibish, i.e. from ~170 ka, according to Brown et al. (2012, ref.[3]). We were unable to locate the Nakaa'kire Tuff at its type section, nor were we able to access samples from the WAVT at Herto. Consequently, our study focuses on geochemical characterisation of the KHS, and identification and dating its proximal counterparts. Important chronostratigraphic control is provided by the characterisation, correlation and dating of the overlying tephra ETH18-8. In addition, accessible tuffs from the Gademotta and Konso formation that appear stratigraphically consistent with the timeframe of interest were sampled and included for comparison.

Methods

All geochemical datasets were initially reviewed to identify any clear outliers arising from either (i) accidental incorporation of a crystal inclusion in the glass analyses, or (ii) glass shards suffering unusually high alkali mobilisation / Na-loss, indicated by Na_2O values < 2 wt% and low totals <91 wt%. Outlier removal was carried out conservatively to prevent accidental removal of shards that might represent true variability in magmatic composition. Marginal outliers were removed from plots, but have been left in Extended Data Table S1 for completeness (marked as *Discarded analyses*). There is evidence for greater glass alteration through alkali loss and hydrogen exchange in all of the distal tuff units, compared to the proximal ignimbrite samples (Figure S3), in line with past studies of Quaternary-age distal peralkaline tuffs in Ethiopia[1,4]. Consequently, Na_2O and K_2O were considered as indicative, not diagnostic in proximal-distal comparisons. Concentrations of P_2O_5 were below or too close to detection limit in all samples and are therefore not reported in Extended Data Table S1.

Geochemical correlations were based on a range of major and trace element bi-plots (Figures 3 and S4), which allow the internal variability of the datasets to be observed and visual assessment of the overlap between samples be made. Principal Component Analysis was run on the major and trace element

datasets (separately) to verify bi-plot interpretations and explore the data in multivariate space (Figure S5). To avoid miscorrelations due to differential alteration, Na_2O and K_2O were removed prior to normalisation for the major element PCA, and the trace element PCA was limited to immobile elements and those comfortably above their limits of detection (Y, Zr, Nb, La, Ce, Nd, Hf, Ta, Th).

The pantelleritic rhyolite nature of the glasses analysed here (Figure 3) as well as their level of alteration leaves only a few immobile major oxide elements useful to differentiate glass compositions. Concentrations of oxides such as CaO and TiO_2 (Figure 3, Extended Data Table S1) are typical of other silicic products of the MER systems (e.g. ref.[1]). Large arrays of immobile trace element abundances within each sample (Figure S4) are a common feature of peralkaline melts. For example, the peralkaline Green Tuff on the island of Pantelleria shows a wide spread of immobile trace element abundances through the c. 7-m-thick eruptive sequence, with Zr varying by 1500 ppm, Nb by 300 ppm and Th by 35 ppm (ref.[5]). Such internal variabilities preclude straightforward correlation on element-element biplots (Figure S4), and therefore we represent trace elements using ratio values to accentuate minor compositional differences and remove the potential effect of concentration variations in the erupted products (Figure 3). This is justified because ratios of immobile trace elements plot as linear arrays and have been shown to be highly effective at distinguishing the different MER tephra and magmatic sources (e.g. refs.[6,7]).

Tephra correlations

In the following, we describe the composition of each sample and discuss their correlations.

The ca. 233 ka Qi2 Shala ignimbrite and KHS tuff

Samples 17-14A1, 17-14B5 and 17-14C from the Qi2 ignimbrite display a homogeneous pantelleritic rhyolitic composition (Table S1). Samples 17-14B5 and 17-14C were analysed for their trace element abundances, whilst sample 17-14A1 did not contain suitable glass shards. Trace element abundances in 17-14C glasses reveal two populations (Table S2, Figure S4). Population 1 plots on the trend line of 17-14B5, while population 2 displays higher contents in all immobile elements with respect to Th, and plots close to the differentiation trend of COI2E/18-8/TA-55 samples, yet without overlapping their composition. This chemical variability may reflect the heterogeneous nature of a melt-mush magmatic system as observed at Aluto[8], with the eruption tapping isolated melt pockets with slightly different mineral phase abundances, resulting in subtle shifts in trace element abundances of the melt.

The KHS glasses form a homogeneous cluster that systematically overlaps the Qi2 samples on major element plots (Figure 3). Apart from slightly higher Nb contents (Figure S4), immobile trace element abundances plot on the trend line of the Qi2 glasses, and overlap with sample 17-14B5. The correlation of the KHS tuff with the Qi2 eruption of Shala is confirmed by principal component analysis of major and trace elements (Figure S5).

We note that the bimodality of 17-14C is not reflected in the KHS tuff. We stress that given the magnitude of the Qi2 eruption, geochemical heterogeneity across the different phases of the eruption is not surprising, and it is likely that tephra from distal exposures may only partially reflect the compositional spectrum of proximal deposits as observed in the Campanian Ignimbrite[9]. This could be due to (i) the erosion of the topmost part of the KHS tuff where co-ignimbrite ash from the last phase of the Qi2 eruption would have deposited[10]; or (ii) a variation in the transport and deposition process of the co-ignimbrite ash of the Qi2 units (i.e. related to eruption intensity, plume height and prevailing wind fields).

The ca. 177 ka Corbetti ignimbrite, Kibish 18-8 tuff and Konso TA-56 tuff

The ca. 177 ka Corbetti ignimbrite (COI2E) is a homogenous pantellerite rhyolite (74.3±0.2 wt% SiO_2, 9.1±0.1 wt% Al_2O_3, 5.6±0.2 wt% FeO*, 10.1±0.2 wt% Na_2O+K_2O, Figure 3, Table S1), displaying some variation in CaO abundances (Figure 3). Immobile trace element abundances in COI2E glasses are systematically higher than those of the Shala Qi2 and KHS glasses with respect to Th (Figure S4).

Glasses from Kibish 18-8 and Konso TA-56 tuffs reveal similar bi-modal compositions on major element biplots (Figure 3, Table S1), with a more differentiated sub-population that overlaps the Shala Qi2/KHS clusters. Immobile trace element abundances of 18-8 and TA-56 samples overlap each other and plot on that of the Corbetti COI2E ignimbrite (Figure S5, Table S2). Figure S5 shows that Konso TA-56 glasses display some more differentiated outliers, while a couple of Kibish 18-8 glass shards are less evolved, yet all the outliers plot on the same differentiation trend. We attribute these compositional ranges to the peralkaline nature of these samples, which show large variations in immobile element concentrations, but limited variation when ratioed (Figure 3)[5].

The compositional overlap between COI2E, 18-8 and TA-56 (Figures 3), confirmed by PCA (Figure S5), indicates that the ca. 177 ka Corbetti ignimbrite is the source of the tuffs at Kibish and Konso. We suggest that the wider compositional range of distal samples from Kibish and Konso records compositional variations within several phases of the 177 ka Corbetti eruption not encompassed in the ignimbrite sample COI2E, which samples only the main phase of the eruption.

The ca. 184 ka Gademotta Unit D

Glasses from Gademotta Unit D plot in a large cluster in immobile major element biplots (Figure 3), which appears close to KHS and TA-56 glasses, yet without clear overlap except in TiO_2 and FeO* abundances. However, TiO_2 concentrations of ~3.5 wt% are very typical of the products of silicic eruptions of the central MER[1]. Immobile trace element abundances in Unit D reveal two glass populations (Table S2, Figure S4). Population 2 of Unit D glasses appears on some plots close to population 2 of sample 17-14C (Figure S4). However, the lack of overlap in major element plots (Figure

3) and the trace element PCA plot (Figure S5) precludes any correlation of unit D with Qi2 unit C or with other samples of the dataset.

References

1. Fontijn, K. *et al.* Contrasting styles of post-caldera volcanism along the Main Ethiopian Rift: Implications for contemporary volcanic hazards. *J. Volcanol. Geotherm. Res.* **356**, 90–113 (2018).

2. Hutchison, W. *et al.* A pulse of mid-Pleistocene rift volcanism in Ethiopia at the dawn of modern humans. *Nat. Commun.* **7**, 1–12 (2016).

3. Brown, F. H., McDougall, I. & Fleagle, J. G. Correlation of the KHS Tuff of the Kibish Formation to volcanic ash layers at other sites, and the age of early Homo sapiens (Omo I and Omo II). *J. Hum. Evol.* **63**, 577–585 (2012).

4. Peccerillo, A. *et al.* Relationships between Mafic and Peralkaline Silicic Magmatism in Continental Rift Settings: a Petrological, Geochemical and Isotopic Study of the Gedemsa Volcano, Central Ethiopian Rift. *J. Petrol.* **44**, 2003–2032 (2003).

5. Williams, R., Branney, M. J. & Barry, T. L. Temporal and spatial evolution of a waxing then waning catastrophic density current revealed by chemical mapping. *Geology* **42**, 107–110 (2014).

6. Hutchison, W. *et al.* The evolution of magma during continental rifting: New constraints from the isotopic and trace element signatures of silicic magmas from Ethiopian volcanoes. *Earth Planet. Sci. Lett.* **489**, 203–218 (2018).

7. Martin-Jones, C. M. *et al.* Recurrent explosive eruptions from a high-risk Main Ethiopian Rift volcano throughout the Holocene. *Geology* **45**, 1127–1130 (2017).

8. Iddon, F. *et al.* Mixing and Crystal Scavenging in the Main Ethiopian Rift Revealed by Trace Element Systematics in Feldspars and Glasses. *Geochemistry, Geophys. Geosystems* **20**, 230–259 (2019).

9. Tomlinson, E. L. *et al.* Geochemistry of the Phlegraean Fields (Italy) proximal sources for major Mediterranean tephras: Implications for the dispersal of Plinian and co-ignimbritic components of explosive eruptions. *Geochim. Cosmochim. Acta* **93**, 102–128 (2012).

10. Cutler, N. A., Streeter, R. T., Dugmore, A. J. & Sear, E. R. How do the grain size characteristics of a tephra deposit change over time? *Bull. Volcanol. 2021 837* **83**, 1–7 (2021).

SUPPLEMENTARY FIGURES

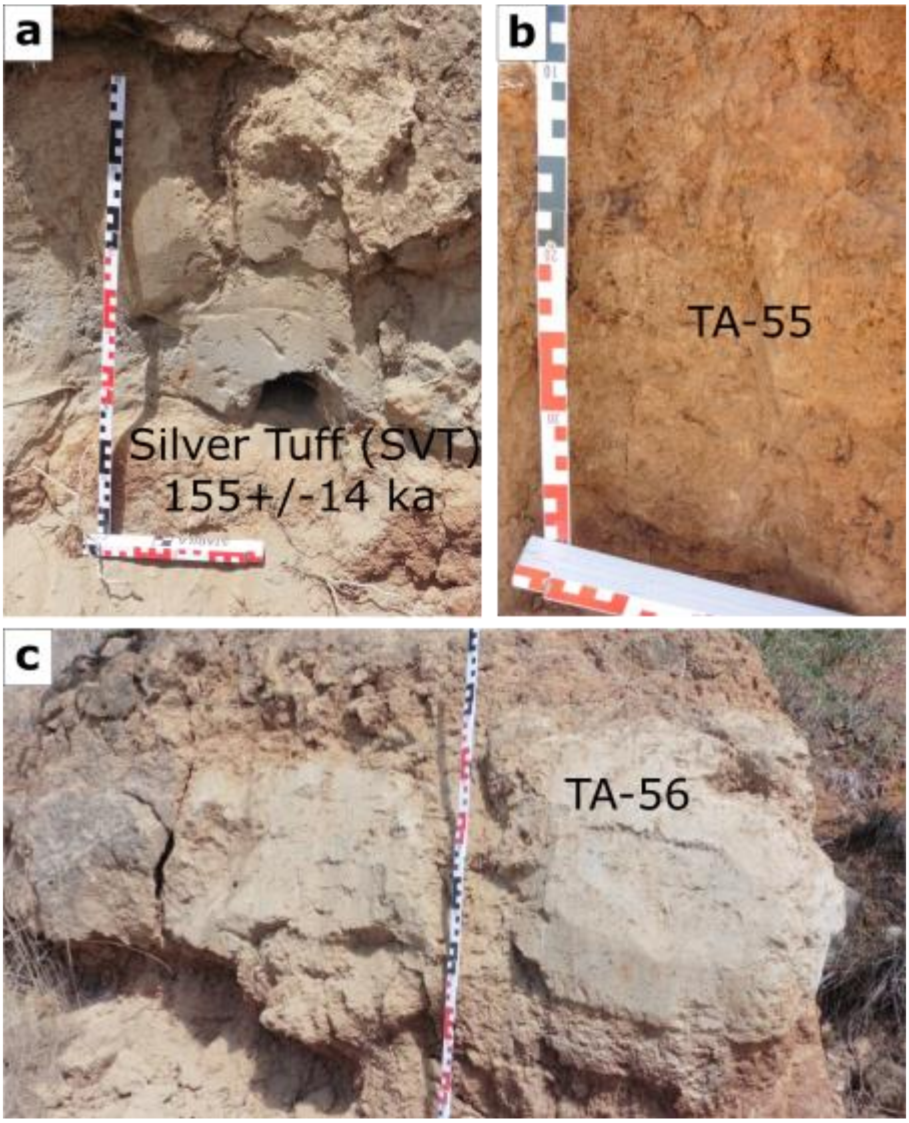

Figure S1. Field photographs of Late Middle Pleistocene tuffs at Konso. See composite stratigraphy in Figure 1b (refs. [1–3])

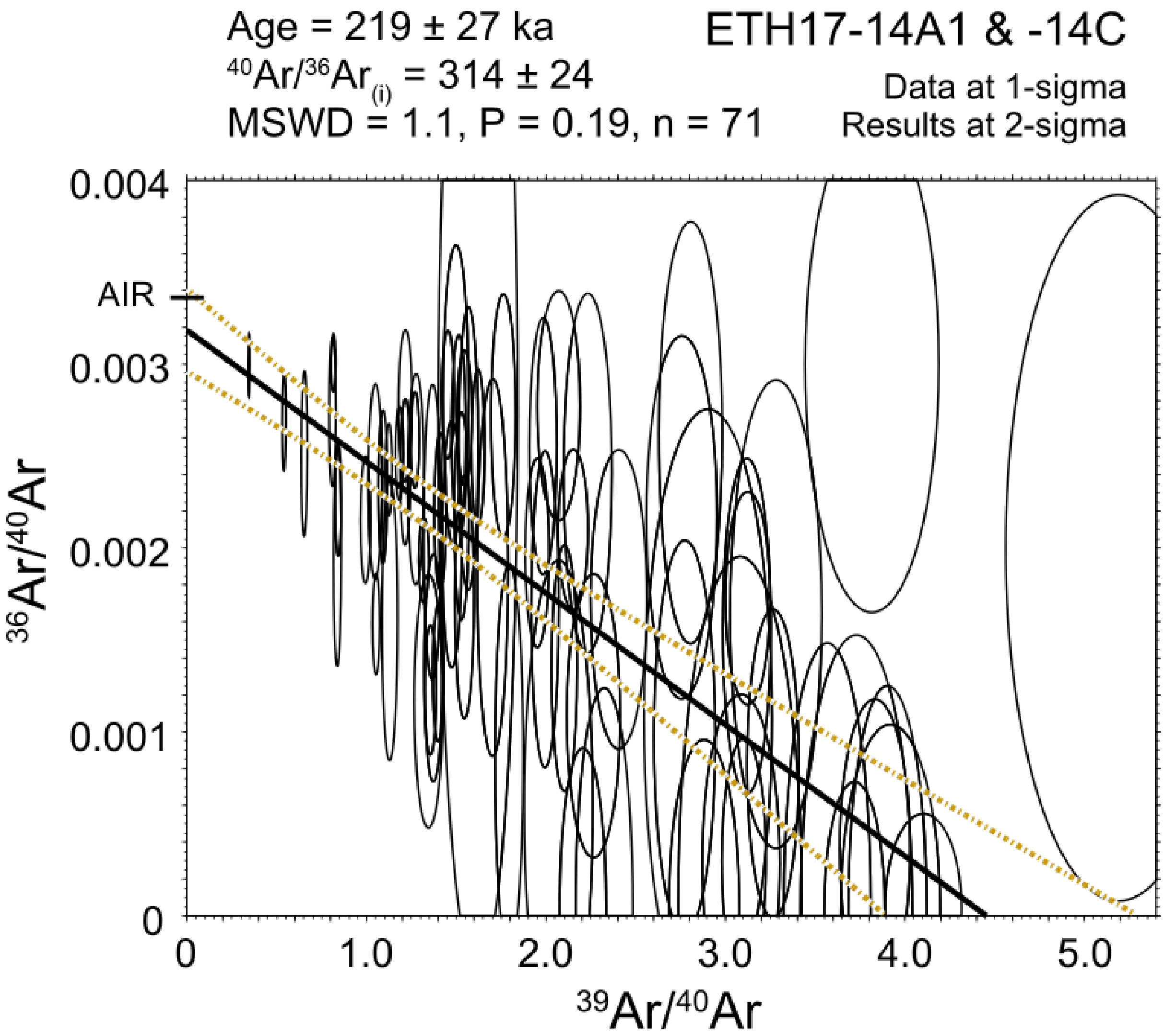

Figure S2. Inverse isochron of data (least squares fit) from samples ETH17-14A1 and ETH17-14C showing regression line and 2σ error envelope. The inverse isochron age is indistinguishable from the accepted age and has a trapped component that is indistinguishable from air suggesting the absence of any excess ^{40}Ar component.

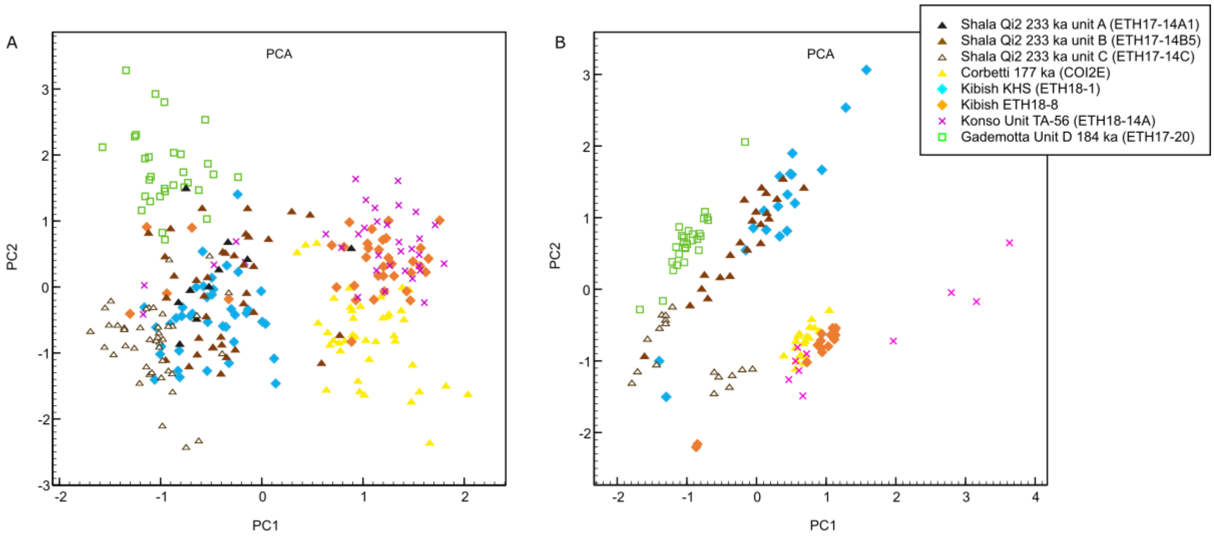

Figure S3. Bi-plots of unnormalised alkali oxides Na_2O (left), K_2O (right) vs analytical totals for tuffs studied in this paper. The correlation between alkalis and analytical total for distal tuffs (those from Gademotta, Kibish and Konso) suggests that glass shards have undergone alkali loss in exchange for hydrogen ions during hydration alteration. Alkali loss is dominated by either Na_2O loss (Gademotta Unit D; Kibish KHS and Konso TA-56), or K_2O loss (Kibish ETH18-8), as described by Cerling et al., (1985, ref.[4]). Proximal ignimbrite samples from Corbetti and Shala do not show the same trends and have a lesser degree of hydration indicated by higher analytical totals. Further differences between analytical totals recorded for different samples may result from unmeasured volatile contents and grain textures (smaller shard sizes and vesicularity can reduce the amount of the EPMA spot encompassed by the sectioned glass shards).

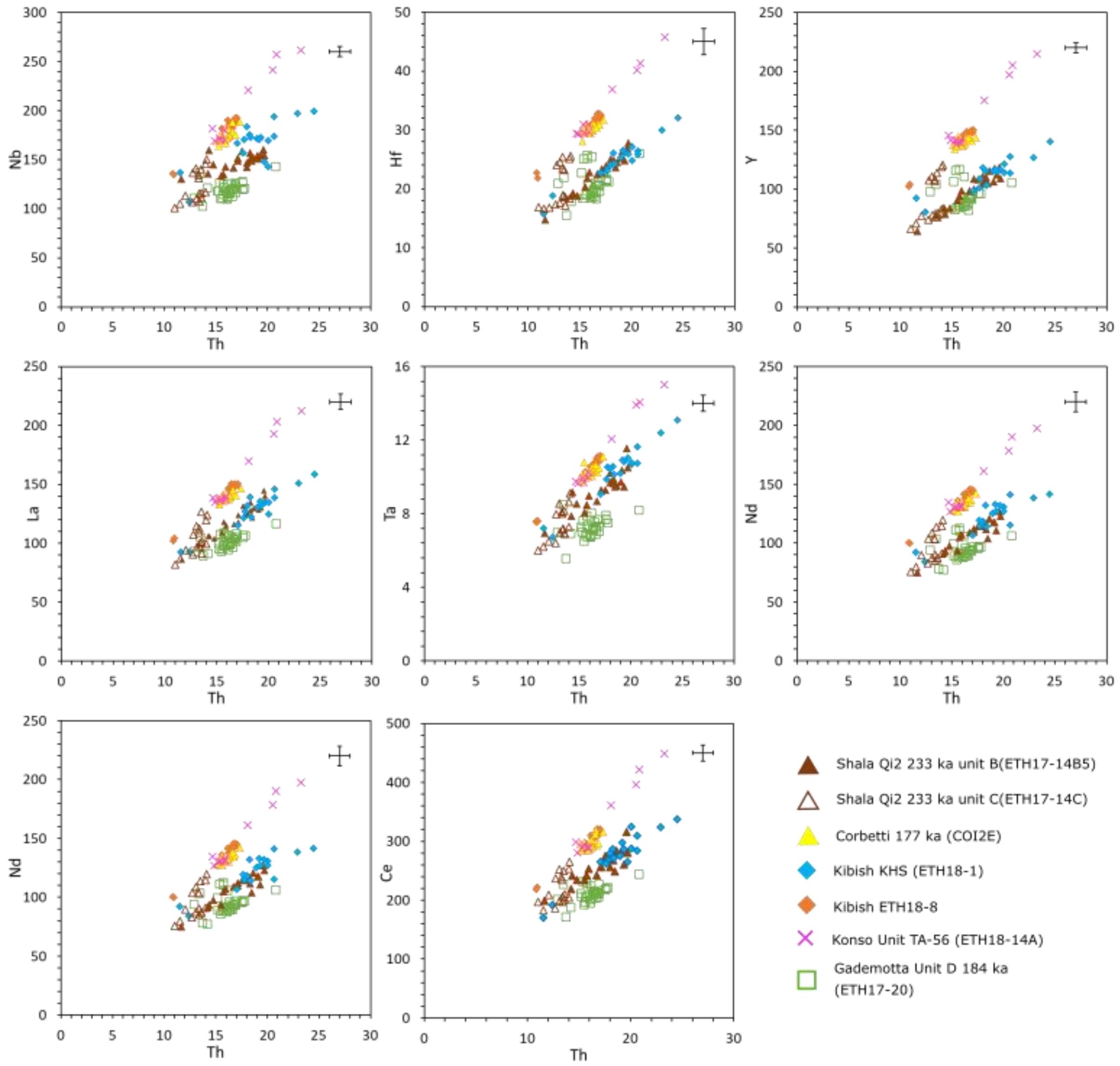

Figure S4. Trace element abundances of glasses from the ca. 233 ka Shala Qi2 ignimbrite, the ca. 177 ka Corbetti ignimbrite, the ca. 184 ka Gademotta Unit D, the Kibish KHS and ETH18-8 tuffs and Konso TA-56 tuffs (all data from this study). Error bars represent one standard deviation of repeated analyses of matrix match glass secondary standard ATHO-G (n=15; Table S7). Error bars shown are relative standard deviations derived from repeat measurements of matrix match glass secondary standard ATHO-G (n=15; Table S7). They are plotted in the upper right corner of each plot for clarity and rescaled to the value of the centre point.

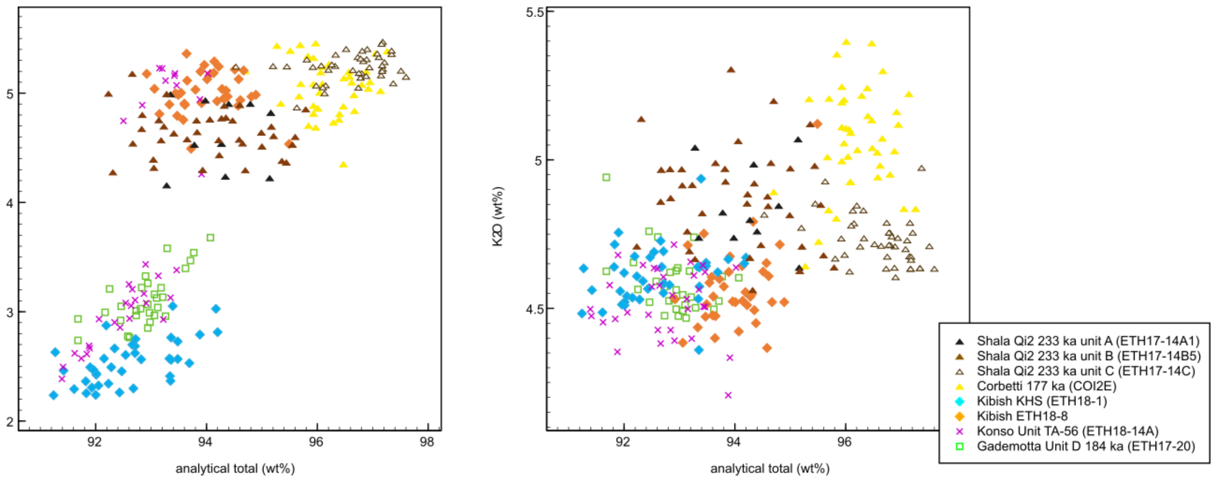

Figure S5. Ordination diagram showing plot of first two principal components of major and minor element oxide data (left, alkalis removed) and immobile trace elements (right), from the tuffs sampled in this paper. Clustering of correlated tuffs is indicative, not perfect, due to the impact of outlier glass shard analyses and geochemical variability within individual datasets, which in some cases comprise <10 hits. PCA and plots generated using the in-built analytical tools within the RESET Database[5].

References

1. Katoh, S. *et al.* Chronostratigraphy and correlation of the Plio-Pleistocene tephra layers of the Konso Formation, southern Main Ethiopian Rift, Ethiopia. *Quat. Sci. Rev.* **19**, 1305–1317 (2000).

2. Nagaoka, S. *et al.* Lithostratigraphy and sedimentary environments of the hominid-bearing Pliocene–Pleistocene Konso Formation in the southern Main Ethiopian Rift, Ethiopia. *Palaeogeogr. Palaeoclimatol. Palaeoecol.* **216**, 333–357 (2005).

3. White, T. D. *et al.* Pleistocene Homo sapiens from Middle Awash, Ethiopia. *Nature* **423**, 742–747 (2003).

4. Cerling, T. E., Brown, F. H. & Bowman, J. R. Low-temperature alteration of volcanic glass: Hydration, Na, K, 18O and Ar mobility. *Chem. Geol. Isot. Geosci. Sect.* **52**, 281–293 (1985).

5. Bronk Ramsey, C., Housley, R. A., Lane, C. S., Smith, V. C. & Pollard, A. M. The RESET tephra database and associated analytical tools. *Quat. Sci. Rev.* **118**, 33–47 (2015).
