# Supplementary material for: Age of the oldest known Homo sapiens from eastern Africa
Source: Nature. 2022 Jan 12;601(7894):579–83. doi: 10.1038/s41586-021-04275-8 (PMC8791829; doi:10.1038/s41586-021-04275-8)
Supplement: Supplementary file 2 — Reporting Summary [file 41586_2021_4275_MOESM2_ESM.pdf]

nature portfolio

Corresponding author(s): Céline Vidal

Last updated by author(s): Oct 22, 2021

Reporting Summary

Nature Portfolio wishes to improve the reproducibility of the work that we publish. This form provides structure for consistency and transparency in reporting. For further information on Nature Portfolio policies, see our Editorial Policies and the Editorial Policy Checklist.

Statistics

For all statistical analyses, confirm that the following items are present in the figure legend, table legend, main text, or Methods section.

n/a	Confirmed	
☐	☒	The exact sample size (*n*) for each experimental group/condition, given as a discrete number and unit of measurement
☐	☒	A statement on whether measurements were taken from distinct samples or whether the same sample was measured repeatedly
☐	☒	The statistical test(s) used AND whether they are one- or two-sided<br>*Only common tests should be described solely by name; describe more complex techniques in the Methods section.*
☒	☐	A description of all covariates tested
☐	☒	A description of any assumptions or corrections, such as tests of normality and adjustment for multiple comparisons
☐	☒	A full description of the statistical parameters including central tendency (e.g. means) or other basic estimates (e.g. regression coefficient) AND variation (e.g. standard deviation) or associated estimates of uncertainty (e.g. confidence intervals)
☐	☒	For null hypothesis testing, the test statistic (e.g. *F*, *t*, *r*) with confidence intervals, effect sizes, degrees of freedom and *P* value noted<br>*Give P values as exact values whenever suitable.*
☒	☐	For Bayesian analysis, information on the choice of priors and Markov chain Monte Carlo settings
☒	☐	For hierarchical and complex designs, identification of the appropriate level for tests and full reporting of outcomes
☒	☐	Estimates of effect sizes (e.g. Cohen's *d*, Pearson's *r*), indicating how they were calculated

Our web collection on statistics for biologists contains articles on many of the points above.

Software and code

Policy information about availability of computer code

Data collection	no software was used
Data analysis	Ar/Ar data was processed using MassSpec, version 8.058, authored by Al Deino, Berkeley Geochronology Center<br>Trace element data reduction was undertaken in Iolite v3.4.

For manuscripts utilizing custom algorithms or software that are central to the research but not yet described in published literature, software must be made available to editors and reviewers. We strongly encourage code deposition in a community repository (e.g. GitHub). See the Nature Portfolio guidelines for submitting code & software for further information.

Data

Policy information about availability of data

All manuscripts must include a data availability statement. This statement should provide the following information, where applicable:

- Accession codes, unique identifiers, or web links for publicly available datasets
- A description of any restrictions on data availability
- For clinical datasets or third party data, please ensure that the statement adheres to our policy

The authors declare that all data supporting the findings of this study are available within the paper and its supplementary information files. The authors declare that all data supporting the findings of this study are available within the paper and its supplementary information files. Background maps for Figure 1 are Shuttle Radar Topography Mission Digital Elevation Model data at one arcsecond resolution from the NASA Land Processes Distributed Active Archive Center (https://earthexplorer.usgs.gov/); settlements, lakes and other features are from (https://www.naturalearthdata.com/). Background image for the top left corner inset of Figure 1 from Google Earth and plate boundaries data courtesy of the U.S. Geological Survey.

March 2021

Field-specific reporting

Please select the one below that is the best fit for your research. If you are not sure, read the appropriate sections before making your selection.

☐ Life sciences ☐ Behavioural & social sciences ☒ Ecological, evolutionary & environmental sciences

For a reference copy of the document with all sections, see nature.com/documents/nr-reporting-summary-flat.pdf

Ecological, evolutionary & environmental sciences study design

All studies must disclose on these points even when the disclosure is negative.

Study description

Geological sampling and geochemical characterisation of volcanic deposits of the Main Ethiopian Rift (MER)

Research sample

We sampled exclusively volcanic desposits mentioned in the litterature that have been used to constrain the age of Omo I fossils. This includes tephra from Konso, Omo-Kibish, and Gademotta formations. We also sampled the previously described Qi2 eruption deposits of Shala volcano. Each sample was collected at its type section described in previous work, were the deposits are the most representative of each unit.

Sampling strategy

We sampled ~ 500g of material for each deposit in clean sample bags for Ar/Ar and geochemical analyses and repository. The quantity collected ensured to display enough grains for dating after cleaning (50-100 grains) and to conduct reproducible analyses of volcanic glass in the appropriate grainsize section, i.e. 40-50 point analyses per sample, following international standards of tephrochronology work.

Data collection

At each site we described extensively the stratigraphy of the outcrops, measured the thickness of units and sampled deposits where best exposed and least altered. Tephra deposits were carefully sampled using a spatula or hammer (on indurated deposits) on cleaned and least altered outcrops. C. Vidal, C. Lane, A. Asrat, A. Deino, G. Yirgu, and A. Zafu collected the samples. C. Vidal,C. Lane, A. Deino and A. Zafu described the deposits (position, texture, thickness, color, grain size, alteration) and their environment and drew stratigraphic sections.

Timing and spatial scale

Fieldwork took place during the Ethiopian dry seasons (spring and autumn). Proximal samples for the Qi2 Shala eruption were sampled in May 2017 at Labusuka village, were it was previously described by Mohr et al. (1980) and where the the exposure of the deposits is maximum in order to sample all units of the eruption. Tephra from Kibish and Konso were sampled in November 2018 during a second mission focused on sampling in southern Ethiopia. These were sampled at their type section, where exposure of the whole deposits is the best.

Data exclusions

No data was excluded in the sampling.
Exclusion strategy for Ar/Ar dating: Following the approach of Kuiper et al. (2008, ref. 19) samples with low radiogenic yields ($40Ar^*<10\%$, 23 grains), and obvious outliers (age > 1Ma, 6 grains) were rejected. Following this initial filtering, peak age distributions were defined by determining the youngest population of individual grain analyses ($n\geq10$) that conforms to a Gaussian distribution with the expected scatter as indicated by the value of MSWD (Mean Square of Weighted Deviates); this second stage of filtering resulted in rejection of an additional ten older grains, leaving 71 accepted grains.
Exclusion strategy for glass analyses: All geochemical datasets were initially reviewed to identify any clear outliers arising from either (i) accidental incorporation of a crystal inclusion in the glass analyses, or (ii) glass shards suffering unusually high alkali mobilisation / Na-loss, indicated by $Na2O$ values < 2 wt% and low totals <91 wt%. Outlier removal was carried out conservatively to prevent accidental removal of shards that might represent true variability in magmatic composition. Marginal outliers were removed from plots, but have been left in Table S1 for completeness (marked as Discarded analyses).

Reproducibility

No experiment were conducted.

Randomization

Randomization is not applicable in the study of volcanic deposits

Blinding

Blinding is not applicable in the study of volcanic deposits

Did the study involve field work? ☒ Yes ☐ No

Field work, collection and transport

Field conditions

Ethiopian Dry season (May 2017 and November 2018). The season was not relevant for the rock samples in particular but favoured the access to remote location by foot/car and bearable heat conditions.

Location

Shala Qi2 deposits in Labusuka (Site ETH17-14): Lat N 7.415018, Long E 38.456357
Konso (site ETH18-14): Lat N 5.41611, Long E 37.36317
Kibish KS type section (site ETH18-8): Lat N 5.34778, Long E 35.93684

Access & import/export

No permit is required for the collection a geological samples in Ethiopia. The sampling of Shala deposits was authorised by the School of Earth Sciences Addis Ababa University and the Oromiya Regional State (24 April 2017). Sampling at Konso and Kibish was permitted by the School of Earth Sciences Addis Ababa University and Ngangatom Woreda Local Administration (15 November 2018). Sample export was granted by the School of Earth Sciences, Addis Ababa University and FDRE Ministry of Mines, Petroleum and Natural Gas.

Disturbance	Sample collection caused no disturbance.<br>Local inhabitants gathered around during the description and sampling of the deposits and interacted with the team in a peaceful way. In more remote areas such as Konso, no disturbance was caused.

Reporting for specific materials, systems and methods

We require information from authors about some types of materials, experimental systems and methods used in many studies. Here, indicate whether each material, system or method listed is relevant to your study. If you are not sure if a list item applies to your research, read the appropriate section before selecting a response.

Materials & experimental systems

n/a	Involved in the study
☒	☐ Antibodies
☒	☐ Eukaryotic cell lines
☒	☐ Palaeontology and archaeology
☒	☐ Animals and other organisms
☒	☐ Human research participants
☒	☐ Clinical data
☒	☐ Dual use research of concern

Methods

n/a	Involved in the study
☒	☐ ChIP-seq
☒	☐ Flow cytometry
☒	☐ MRI-based neuroimaging
